# Supplementary material for: Nitrogen fixation sustained productivity in the wake of the Palaeoproterozoic Great Oxygenation Event
Source: Nat Commun. 2018 Mar 7;9:978. doi: 10.1038/s41467-018-03361-2 (PMC5841411; doi:10.1038/s41467-018-03361-2)
Supplement: Supplementary file 1 — Supplementary Information [file 41467_2018_3361_MOESM1_ESM.pdf]

Supplemental Information

Luo et al.

Nitrogen fixation sustained productivity in the wake of the Paleoproterozoic Great Oxygenation Event

Supplementary Table 1. Tabulated data from the cores analyzed in this study. Data have been derived from bulk decarbonated residues and kerogen extracts. HF insoluble heavy minerals were not removed from the kerogen isolates, which is reflected in the relatively low purity kerogen isolates. This does not affect the isotope data that are expressed relative to their respective delta zeros. Elemental ratios are expressed as atomic ratios (*) and shaded cells reflect no data. Lithostratigraphy and placement of the formation boundaries, along with the S-isotope stratigraphy (S-Int) follows Luo et al., (2016). S-MIF and S-MDF are mass-independent and mass-dependent sulphur isotope fractionation, respectively.

Fm.	S-Int	Sample Number	Depth (m)	Bulk Measurement					Kerogen				
				TOC (%)	TN_{bulk} (ppm)	C_{org}/N_{bulk}*	$\delta^{13}C_{org}$ (‰, VPDB)	$\delta^{15}N_{bulk}$ (‰, Air)	C_{ker} (%)	N_{ker} (ppm)	$(C/N)_{ker}$*	$\delta^{15}N_{ker}$ (‰, Air)	$HF_{residue}$ (%)
KEA-4													
upper Timeball Hill	S-MDF	KEA-4 216.45-216.64	216.55	2.66	796	39.0	-31.3	2.9					
		KEA-4 217.98-218.04	218.01	2.63	725	42.3	-31.3	2.5	20.1	1900	123.6	2.7	12.95
		KEA-4 219.11-219.23	219.17	2.58	774	38.9	-31.3	3.2					
		KEA-4 225.23-225.32	225.28	4.3	680	73.8	-31.7	2.5	24.1	570	494.1	2.0	

		KEA-4 226.84-226.91	226.88	1.42	678	24.4	-31.3	2.8	56.1	2000	327.4	0.5	
		KEA-4 229.72-229.84	229.78	1.73	775	26.1	-31.9	3.1					
		KEA-4 234.73-234.84	234.78	4.1	655	73.1	-31.4	2.8	54.8	2140	298.8	1.0	3.77
		KEA-4 237.08-237.16	237.12	7.1			-31.5						
lower Timeball Hill		KEA-4 421.76-421.85	421.81	0.49	282	20.3	-33.3	7.0	3.3	150	252.8	7.8	3.94
		KEA-4 428.59-428.72	428.65	0.22	267	9.6	-31.7	6.4					
		KEA-4 434.08-434.31	434.2	0.24	319	8.8	-32.8	7.0					
		KEA-4 450.72-450.83	450.78	0.49	302	18.9	-32.7	6.7					
		KEA-4 451.33-451.51	451.42	0.2	218	10.7	-31.5	6.8					
		KEA-4 466.92-467.04	466.98	0.47	402	13.6	-32	7.1	3.2	480	77.0	7.7	7.48
		KEA-4 478.84-478.96	478.9	0.64	389	19.2	-32.5	7.6	5.2	530	115.1	7.4	7.35
		KEA-4 482.04-482.20	482.12	0.78	381	23.9	-32.2	6.9	4.7	410	133.7	6.1	10.26
		KEA-4 486.20-486.33-B	486.26	0.56	384	17.0	-32.1	7.6					
		KEA-4 486.20-486.33-A	486.26	0.57	403	16.5	-32.3	7.3	31.8	1250	297.0	5.3	0.81

		KEA-4 492.44-492.63	492.54	0.69	402	20.0	-31.8	7.5						
		KEA-4 496.79-496.92	496.85	0.98	423	27.0	-32.3	7.5						
		KEA-4 498.22-498.31	498.27	0.61	411	17.3	-31.9	7.6						
		KEA-4 515.63-515.74	515.69	0.87	435	23.3	-33.2	7.5						
		KEA-4 519.56-519.72	519.64	0.74	401	21.6	-33.8	7.2						
		KEA-4 520.64-520.70	520.68	0.98	448	25.5	-33.7	8.5	46.0	2080	257.9	6.1	1.33
		KEA-4 521.51-521.63-D	521.57	1.03	520	23.1	-33.4	7.8						
		KEA-4 521.51-521.63-C	521.57	0.69	508	15.9	-32.9	7.7						
		KEA-4 521.51-521.63-B	521.57	0.36	492	8.5	-32.1	8.2						
		KEA-4 521.51-521.63-A	521.57	0.86	505	19.9	-33.3	7.5						
		KEA-4 527.98-528.06	528.02	0.98	456	25.1	-32.8	7.8	9.4	1130	97.5	8.1	7.62
		KEA-4 535.86-535.98	535.92	0.90	455	23.1	-32.5	7.7	8.7	1120	90.9	8.0	
		KEA-4 548.20-548.76	548.48	3.45	623	64.6	-33.6	8.7						
		KEA-4 548.74-548.96-B	548.85	5.56	691	93.9	-35.7	8.2	78.4	2920	313.3	5.7	5.2

		KEA-4 548.74-548.96-A	548.85	5.78	619	108.9	-35.2	8.0					
		KEA-4 548.96-549.05	549.00	7.16	712	117.4	-34.7	7.7					
		KEA-4 549.31-549.43	549.37	1.45	770	22.0	-33.0	8.7	17.51	1510	135.3	7.8	7.31
		KEA-4 551.04-551.13-B	551.08	0.43	1388	3.6	-32.6	8.8					
		KEA-4 551.04-551.13-A	551.08	6.94	646	125.4	-35.0	7.5					
upper Rooihoogte		KEA-4 553.40-553.54	553.47	5.63	469	140.1	-34.9	7.4					
		KEA-4 553.86-553.98-F	553.92	4.47	432	120.8	-34.9	7.3					
		KEA-4 553.86-553.98-D	553.92	5.36	374	167.2	-35.1	6.5					
		KEA-4 553.86-553.98-C	553.92	4.52	375	140.8	-35.1	7.1	17.0	2210	89.5	5.7	2.1
		KEA-4 553.86-553.98-B	553.92	5.97	462	150.8	-35.4	6.9	17.5	2260	90.3	5.6	
		KEA-4 553.86-553.98-A	553.92	1.96	643	35.6	-35.1	7.5					
		KEA-4 554.38-554.56	554.47	3.00	336	104.1	-34.9	7.7					
		KEA-4 555.85-556.30	556.07	0.65			-33.0						
		KEA-4 556.80-556.90	556.85	0.92	180	59.6	-35.3	7.9	55.6	2440	265.7	9.2	

		KEA-4 558.52-558.60	558.56	2.55	217	137.3	-33.9	6.0						
	Transitional	KEA-4 560.30-560.57	560.47		175		-27.5	7.8						
		KEA-4 561.05-561.14-C	561.10	0.51	350	17.0	-32.4	6.7						
		KEA-4 561.45-561.60-C	561.50	0.39	304	15.0	-33.7	6.7	8.9	520	199.9	4.7		
		KEA-4 561.45-561.60-D	561.53	0.33	300	12.8	-33.1	6.2						
		KEA-4 562.78-562.87-C	562.82	0.31	206	17.6	-33.8	6.2	24.1	940	299.5	4.0		
	S-MIF	KEA-4 563.63-563.73	563.68	0.31	297	12.2	-33.1	6						
		KEA-4 564.24-564.42-A	564.30	0.35	391	10.4	-33.1	6.2	5.6	290	224.5	3.8		
		KEA-4 564.24-564.42-B	564.28	0.55	398	16.1	-33.3	6.6						
		KEA-4 564.24-564.42-B-	564.33	0.16	322	5.8	-32.8	6.4	11.8	620	221.3	3.8	0.27	
		KEA-4 564.74-564.86	564.79	0.49	415	13.8	-33.5	6.1	14.4	640	262.9	3.4		
		KEA-4 565.70-565.84	565.77	0.27	423	7.4	-32.4	6.1	3.3	140	275.8	4.5	0.41	
		KEA-4 565.84-566.0-B	565.90	0.18	381	5.5	-32.6	6.2						
		KEA-4 565.84-566.0-A	565.92	0.24	475	5.9	-32.0	6.4	11.08	520	248.6	3.1		

		KEA-4 566.06-566.10	566.08	0.40	468	10.0	-31.0	6.3	5.1	360	166.6	2.6	
EBA-2													
upper Timeball Hill	S-MDF	EBA-2 809.2	809.20						4.66	70	776.7	3.5	4.7
		EBA-2 868	868.00						81.88	2340	408.2	1.8	0.3
		EBA-2 885.9	885.90						52.67	1500	409.7	1.0	0.3
		EBA-2 910.1	910.10						87.93	2270	451.9	0.8	0.2
		EBA-2 927.1	927.10						11.42	470	283.5	2.9	3.8
		EBA-2-947.6	947.60						84.52	2120	465.1	-1.2	1.0
		EBA-2 981.8	981.80						81.33	3360	282.4	1.0	0.4
		EBA-2 1000	1000.00						50.29	2140	274.2	1.1	0.3
		EBA-2 1027	1027.00						30.3	1410	250.7	1.6	0.0
lower Timeball Hill		EBA-2 1196	1196.00						4.69	350	156.3	5.8	4.2
		EBA-2 1215.5	1215.50						6.14	190	377.0	5.3	3.5
		EBA-2 1252.1	1252.10						8.55	240	415.6	4.5	2.9
		EBA-2 1272.5	1272.50						18.24	640	332.5	4.9	3.3
		EBA-2 1286.1	1286.10						11.78	430	319.6	5.3	4.3
		EBA-2 1301.3	1301.30						17.18	700	286.3	4.3	5.4
		EBA-2 1313.5	1313.50						10.68	360	346.1	4.1	6.3
upper Rooihoogte		EBA-2 1337.29	1337.29	1.40	404	40.4	-34.2	8.2	18.39	800	268.2	5.1	6.0
		EBA-2 1338.28	1338.28	3.10	386	93.8	-34.4	7.7					
		EBA-2 1338.78	1338.78	0.70	385	21.2	-33.3	7.8	9.97	570	204.1	6.8	4.9
	Transitional	EBA-2 1340.16-A	1340.16	0.21	322	7.6	-32.9	6.9	8.07	410	229.6	4.5	

		EBA-2 1340.6-B	1340.16	0.10	448	2.6	-32.9	6.5					
		EBA-2 1340.88	1340.88	0.10	349	3.3	-33.7	6.5	7.7	400	223.7	4.6	0.6
		EBA-2 1341.77-A	1341.77	0.10	312	3.7	-32.0	6.3	30.62	1500	238.2	3.0	
		EBA-2 1341.77-B	1341.77	0.10	445	2.6	-31.2	6.8					
		EBA-2 1341.77-C	1341.77	0.80	312	30.0	-33.6	6.4					
	S-MIF	EBA-2 1343.25	1343.25	0.28	523	6.2	-34.4	6.5					
		EBA-2 1344.56-A	1344.56	0.40	594	7.9	-34.6	6.7	11.43	620	215.1	4.1	
		EBA-2 1344.56	1344.56	0.10	588	2.0	-33.7	6.6					
		EBA-2 1345.18-A	1345.18	0.45	327	16.1	-34.2	6.5					
		EBA-2 1345.18-B	1345.18	0.23	408	6.6	-34.0	6.3					
		EBA-2 1345.18-A(1)	1345.18					6.2					
		EBA-2 1345.48-B	1345.48	0.25	455	6.4	-32.5	6.1	26.08	1030	295.4	3.9	
		EBA-2 1345.48-A	1345.48	0.30	774	4.5	-32.8	6					
		EBA-4											
upper Timeball Hill	S-MDF	EBA-4 573.7	573.70						4.04	350.0	134.7	3.9	2.15
		EBA-4 586.1	586.10						4.95	510.0	113.2	4.2	1.93
		EBA-4 599.6	599.60						11.58	550.0	245.6	4.5	6.52
		EBA-4 605	605.00						81.89	3320.0	287.8	3.6	0.16
		EBA-4 658.5	658.50						41.15	1630.0	294.5	2.2	0.16
		EBA-4 690.7	690.70						3.9	270.0	168.5	3.4	8.73
		EBA-4 702.7	702.70						6.39	480.0	155.3	2.1	
		EBA-4 713.8	713.80						68.83	2920.0	275.0	2.6	0.13
		EBA-4 725.2	725.20						53.66	2390.0	261.9	1.6	0.37

		EBA-4 749.1	749.10						10.2	650.0	183.1	-0.8	4.73
		EBA-4 762.5	762.50						10.18	810.0	146.6	-1.9	3.46
		EBA-4 844.1	844.10						2.03	250.0	94.7	4.7	2.9
lower Timeball Hill		EBA-4 922.1	922.10						2.84	200.0	165.7	2.8	3.34
		EBA-4 930.1	930.10						2.36	200.0	137.7	5.4	4.3
		EBA-4 976.5	976.50						11.9	340.0	408.3	3.6	1.9
		EBA-4 1027.8	1027.80						30.3	1050.0	336.7	3.5	1.66
		EBA-4 1049.5	1049.50						11.76	390.0	351.8	3.9	6.61
		EBA-4 1050.7	1050.70						15.48	800.0	223.0	6.6	6.2
		EBA-4 1071.16-1071.26-A	1071.21	5.14	572	104.8	-35.3	7.7	27.29	1980.0	160.8	7.0	0.54
		EBA-4 1072.05-1072.20	1072.13	5.62	735		-35.1	8.8					
upper Rooihoogte		EBA-4 1072.20-1072.50-A	1072.35		357		-35.3	6.9					
		EBA-4 1072.96-1073.13-UPPER-A	1073.05	3.14	425	86.1	-35.1	7.9	52.81	2030.0	303.5	6.5	5.28
		EBA-4 1074.31-1074.44	1074.38	2.49	392	74.2	-34.3	7.8	17.32	560.0	360.8	5.6	10.39
		EBA-4 1075.26-1075.33	1075.30	0.56	193	33.8	-32.3	7.3	3.2	220.0	169.2	6.8	7.23
		EBA-4 1076.18-1076.41-A	1076.30	0.03	343		-29.1	6.7					
	Transitional	EBA-4 1076.18-1076.41-MIDDLE-C	1076.30	0.21	246	10.0	-31.3	6.7					
		EBA-4 1077.85-1078.02	1077.94	0.24	527	5.3	-33.8	6.7	3.41	330.0	120.6	4.9	0.26
		EBA-4 1078.49-1078.80-UPPER-B	1078.64		460			6.5					
		EBA-4 1078.49-1078.80-A	1078.64	0.16	545	3.4	-31.8	6.6					
		EBA-4 1079.69-1079.91-A	1079.80	0.23	393	6.8	-33	6.8	1.19	120.0	115.7	4.8	
	S-MIF	EBA-4 1080.41-1080.74-upper-d	1080.50	0.21	439	5.6	-34.7	7.2	2.11	300.0	82.1	4.4	
		EBA-4 1080.41-1080.74-UPPER-C	1080.55	0.38	444	10.0	-34.5	6					

		EBA-4 1080.41-1080.74-LOWER-B	1080.60	0.23	471	5.7	-34.3	6.6					
		EBA-4 1080.41-1080.74-LOWER-A	1080.65	0.51	518	11.5	-34.9	6.5					
		EBA-4 1082.36-1082.53	1082.45	0.3	418	8.4	-33.8	7.7	0.93	1800.0	60.3	4.5	11.61
		EBA-4 1083.12-1083.24	1083.18	0.37	408	10.6	-34.8	7.6	3.03	200.0	176.8	1.9	0.78
